# Supplementary material for: Effect of Collagen Matrix on Doxorubicin Distribution and Cancer Cells’ Response to Treatment in 3D Tumor Model
Source: Cancers (Basel). 2022 Nov 8;14(22):5487. doi: 10.3390/cancers14225487 (PMC9688511; doi:10.3390/cancers14225487)
Supplement: Supplementary file 1 [file cancers-14-05487-s001.zip › cancers-1918802-supplementary.pdf]

Single cell FLIM analysis

Fluorescence decay parameters obtained for individual cells in the FLIM images comprised the dataset used to analyze the dynamics of cells response to treatment (Figure S1). Visualization of the FLIM parameters distribution was performed with box plots calculated with the seaborn library (Python 3.1). For the co-culture models, fibroblasts labeled with fluorescent dye (i.e. cells with extremely high fluorescence intensity on the FLIM images) were removed from the dataset.

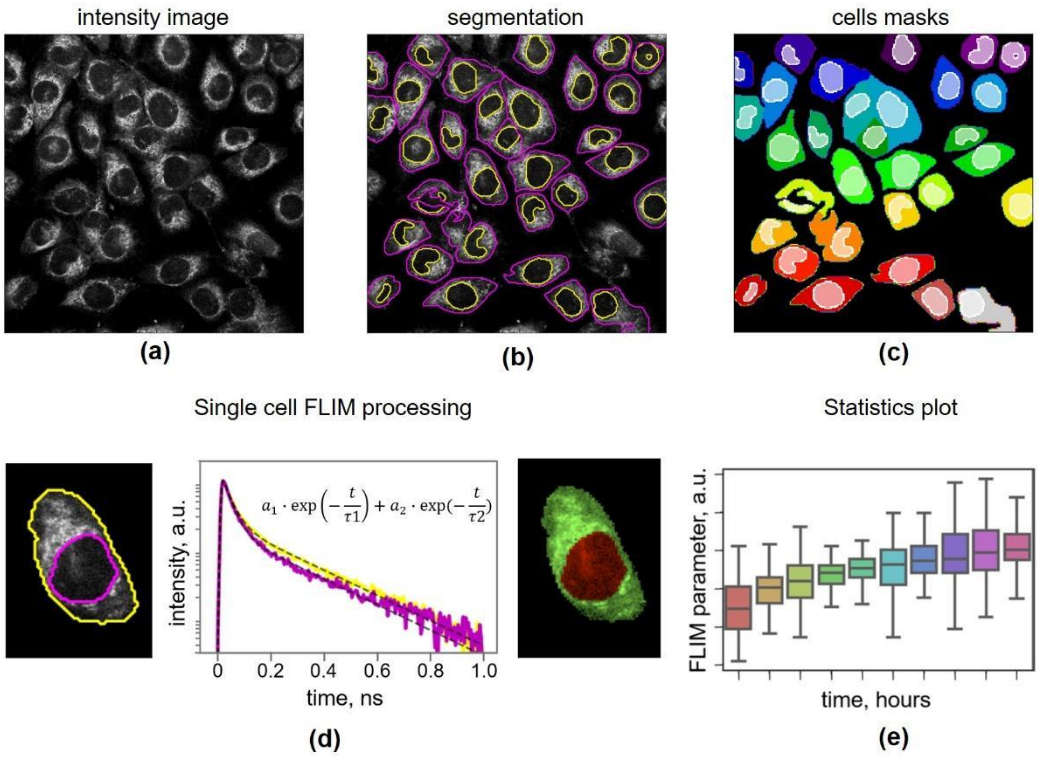

Figure S1. Pipeline of the FLIM data analysis. (a) Fluorescence intensity image of cells. (b) Segmentation of the fluorescence intensity image: determination of contours for cells and nuclei. (c) Masks for individual cells cytoplasm and nuclei. (d) Fitting the FLIM data for individual cells with the exponential decay model. (e) Analysis of the FLIM parameters distribution obtained for individual cells and assessment of the metabolic state.

IC50 determination

The results of the routine MTT assay are shown in Figure S2. The IC50 concentration of DOX for T24 cells was determined to be 51±4 µg/ml and used in all the experiments.

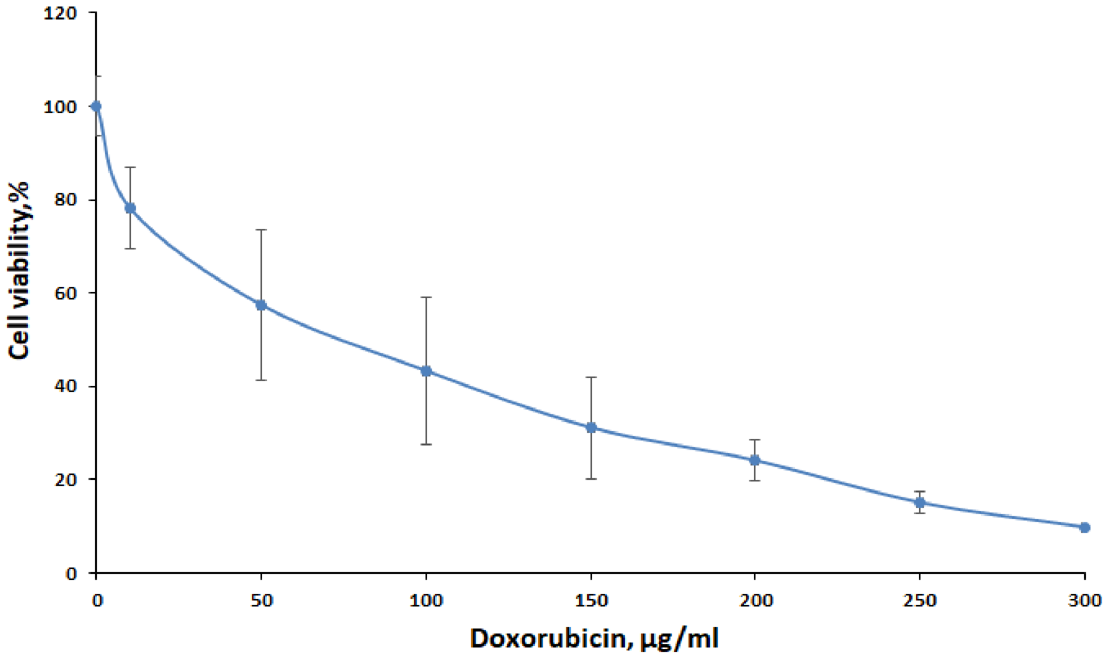

Figure S2. T24 cells viability under DOX exposure determined by MTT assay. Mean±SD, n=3 repetitions by 10 independent measurements.

Monitoring of collagen structure

The collagen structure was analyzed in T24 monoculture and co-culture with fibroblasts during 5 days of cultivation. The representative SHG images in Figure S3 demonstrate the absence of collagen fibers in T24 monoculture up to 24 hours of cultivation; starting from day 2 a weak SHG signal is observed. In the model with fibroblasts, a dense fibrillar structure of collagen was formed already by 24 hours and then did not change.

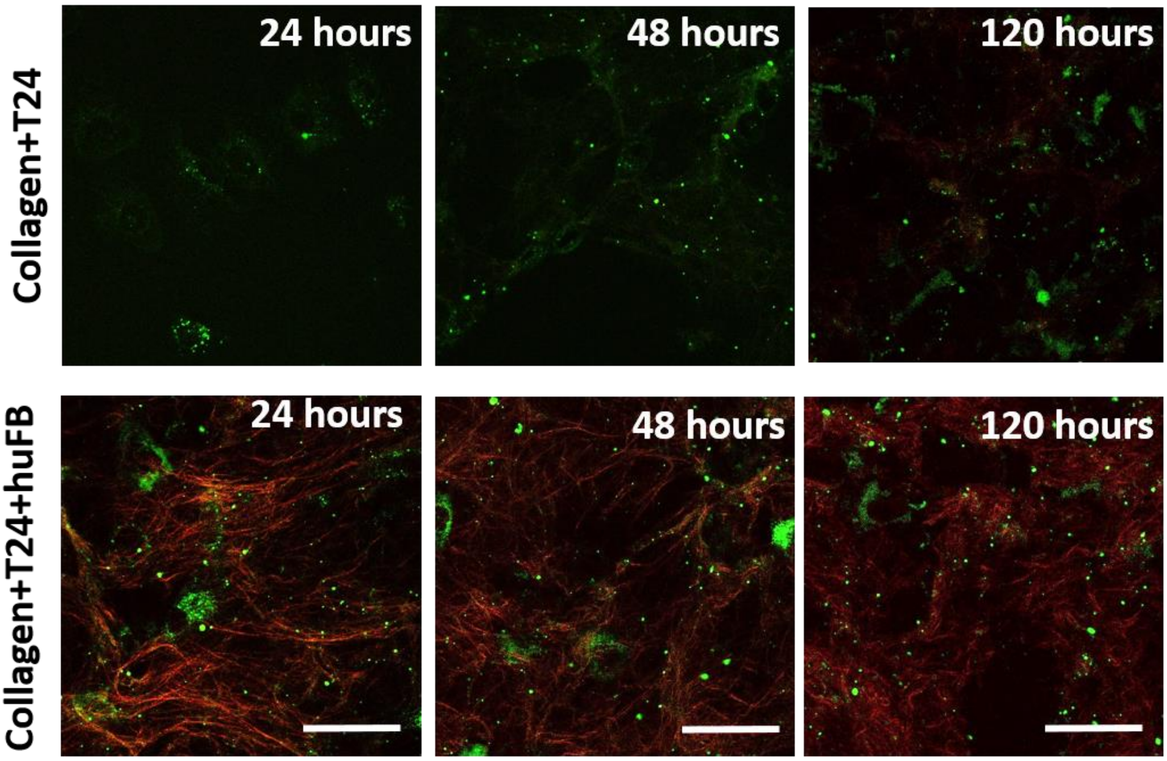

Figure S3. Representative SHG images of collagen in T24 monoculture and co-culture with fibroblasts captured at different times of cultivation. Bar is 50 μm, applicable to all images.

Effect of collagen on DOX delivery to HT29 cancer cells in MFC

Besides T24 cells, human colorectal cancer cells HT29 were used in a 3D model to analyze DOX distribution in MFCs loaded with collagen. Compared to T24 cells that were unable to structurize collagen gel, HT29 cells formed thin collagen fibers (Figure S4a). As a result, DOX reached HT29 cancer, both in monoculture and in co-culture with fibroblasts, faster compared to the corresponding models with T24 cells (Figure S4b). Metabolic imaging using NAD(P)H FLIM revealed lower value of the mean lifetime and greater contribution of NAD(P)H in HT29 cells growing in collagen matrix, compared to the control cells indicating a shift to glycolysis (Figure S4c).

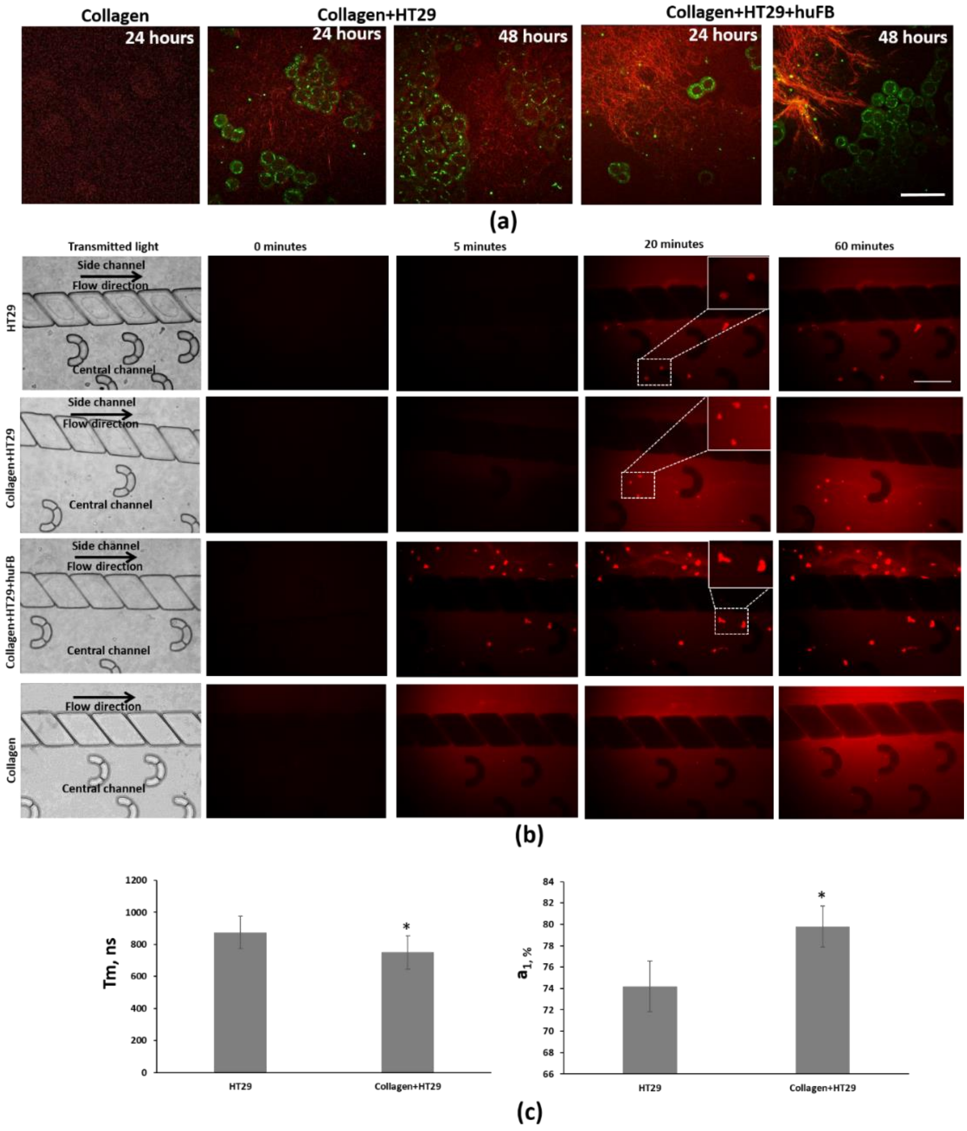

Figure S4. Modeling of DOX delivery to cancer cells in the presence of collagen using MFCs. (a) Representative SHG images of collagen seeded with HT29 cancer cells or co-culture of HT29 and human fibroblasts huFB or without any cells. Red - SHG signal from collagen, green - autofluorescence of cells. Scale bar: 50 μm, applicable to all images. (b) Images of MFC in transmitted light and representative time-lapse images of DOX fluorescence in different models. The areas of DOX uptake into HT29 cells are shown in the dashed squares and enlarged in the right upper corner of the images. Red – Fluorescence of DOX. Scale bar: 400 μm, applicable to all images. (c) FLIM of NAD(P)H in HT29 cells in the absence and presence of collagen. Mean fluorescence lifetime of NAD(P)H τ_m and relative contribution of free NAD(P)H a_1 in the cell cytoplasm. Mean±SD, n=25-30 cells. *Statistically significant difference from HT29 cells without collagen.

Colocalization analysis of DOX and NAD(P)H

Inside the cells DOX fluorescence was observed in both the cell cytoplasm and the nuclei. Colocalization analysis was performed using ImageJ plugin JaCop. The measurement of colocalization of DOX with mitochondria identified by fluorescence of NAD(P)H showed a weak overlap of the signals (the Manders' overlap coefficient M1 0.297, the Pearson correlation 0.186). Representative fluorescence intensity images of DOX and NAD(P)H in T24 cells and a merged image are shown in Figure S5)

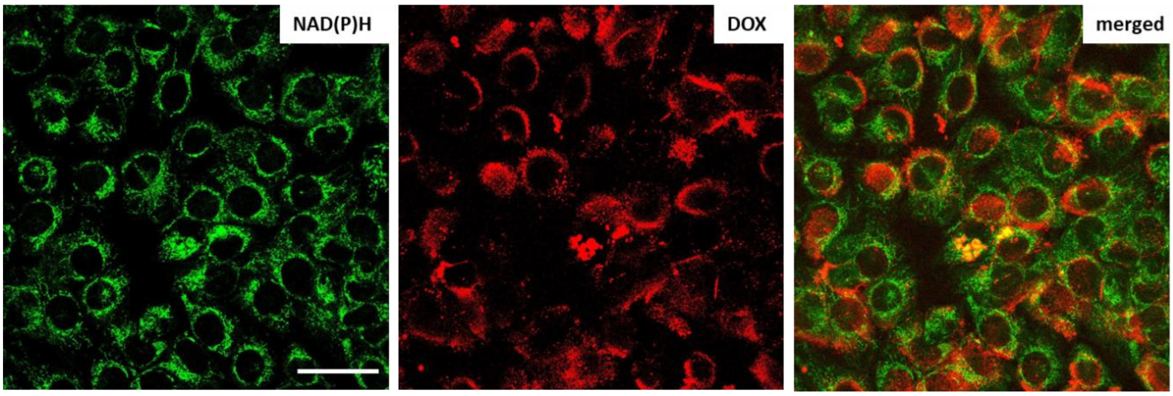

Figure S5. Colocalization analysis of DOX with mitochondrial NAD(P)H. For DOX: ex. 488 nm, reg. 540-650 nm. For NAD(P)H: ex. 750 nm, reg. 455–500 nm. Scale bar is 50 μm.

Effects of low dose of DOX on T24 cells

The dynamics of fluorescence lifetime of NAD(P)H in the cytoplasm and the nuclei of T24 cells after adding a low (IC50/2) dose of DOX in comparison with IC50 dose is presented in Figure S6A. Analysis of DOX fluorescence lifetime and intensity for the two drug doses is shown in Figure S6B,C.

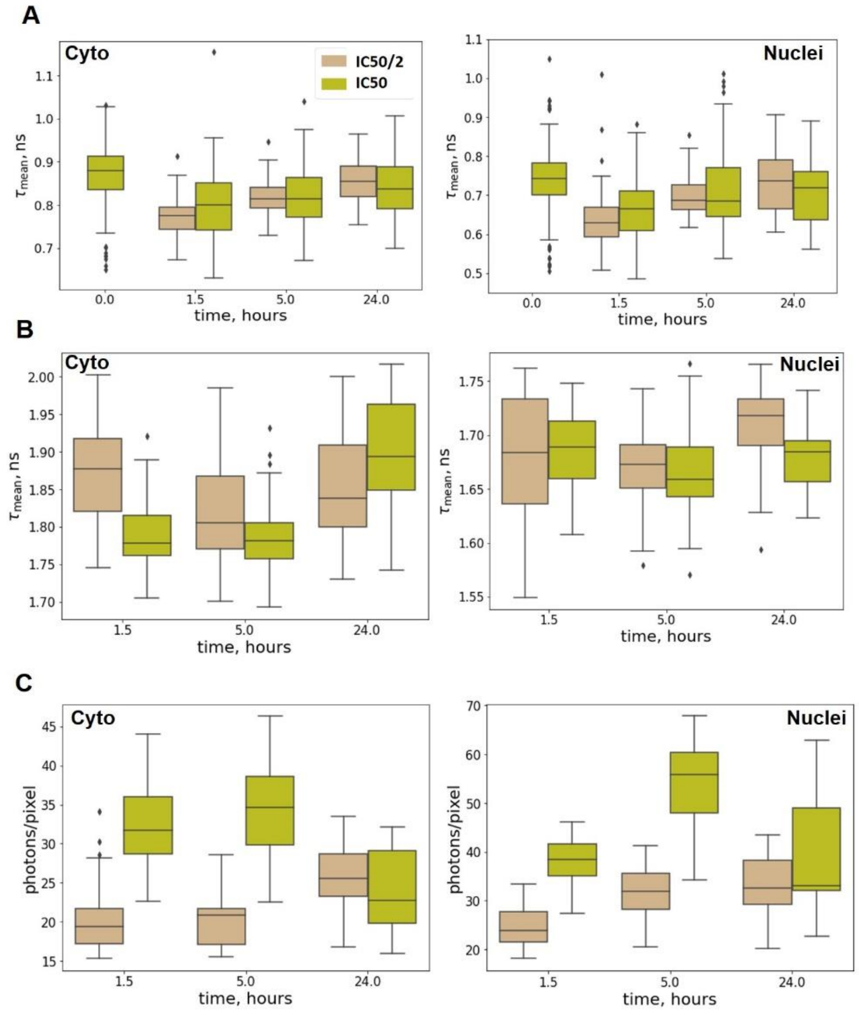

Figure S6. Fluorescence intensities and lifetimes of NAD(P)H and DOX in T24 cells upon treatment with reduced dose of DOX. (a) Analysis of fluorescence lifetimes of NAD(P)H in the cell cytoplasm and in the nuclei. Boxes are quartiles, whiskers are minimums and maximums. (b) Analysis of fluorescence lifetimes of DOX in the cell cytoplasm and in the nuclei. Boxes are quantiles, whiskers are minimums and maximums. (c) Analysis of fluorescence intensity of DOX in the cell cytoplasm and in the nuclei. Boxes are quartiles, whiskers are minimums and maximums. Concentration of DOX was 25 μg/ml (IC50/2).

NAD(P)H FLIM of T24 cells under Doxorubicin exposure

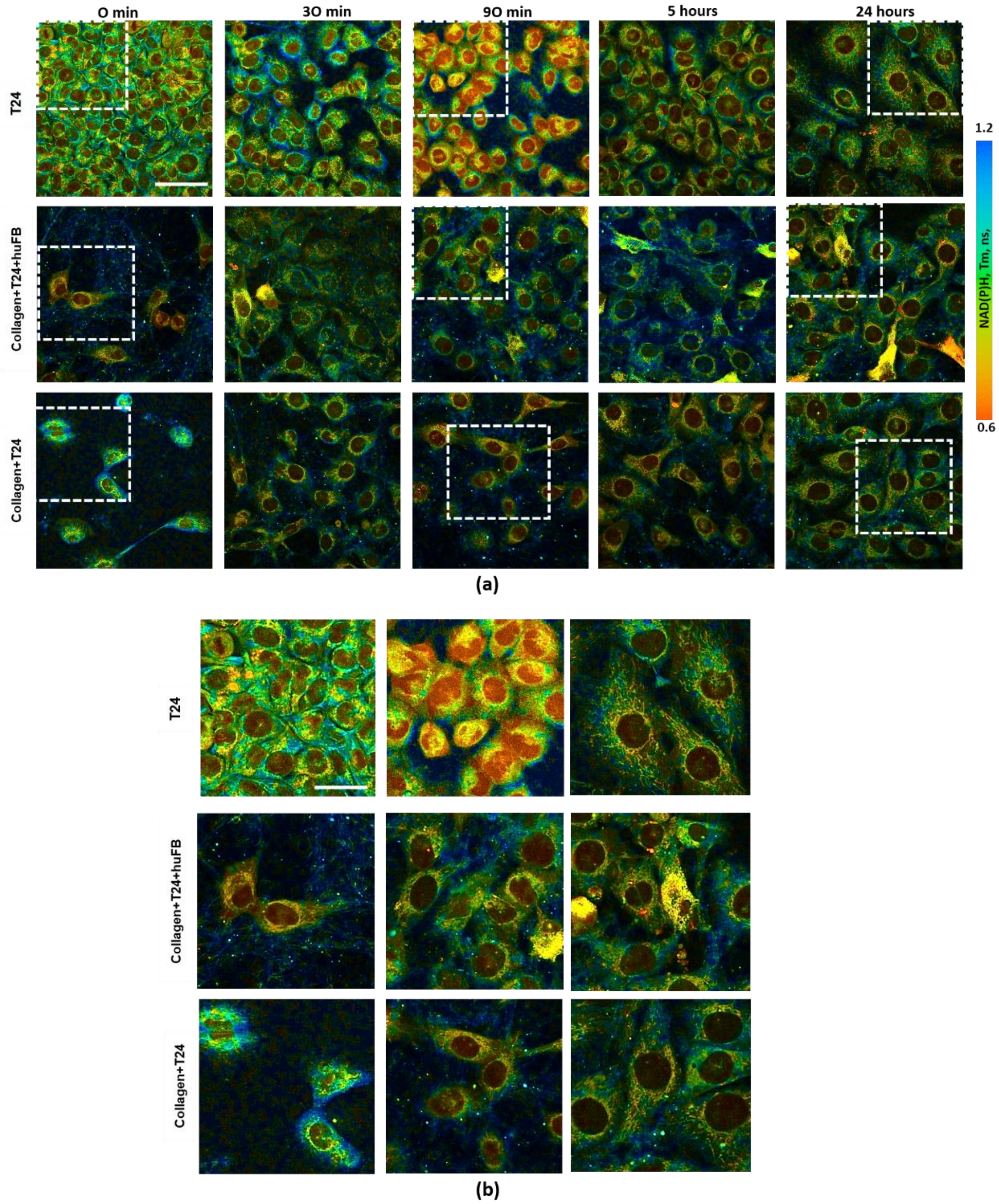

Figure S7. Fluorescence lifetime imaging of NAD(P)H in T24 cells in the absence and presence of collagen. (a) Representative FLIM images extracted from SPCImage software (Becker&Hickl, Germany). Time of incubation with DOX is indicated on the images. Excitation: 750 nm, registration: 455–500 nm. The areas in the dashed squares are presented enlarged in (b). Scale bar is 50 µm, applicable to all images. Concentration of DOX was 50 µg/ml (IC50). (b) Higher magnification images. Scale bar is 25 µm, applicable to all images.

Table S1. NAD(P)H FLIM of T24 cells under DOX exposure. Median values of NAD(P)H FLIM parameters.

Model	time, hours	a1 nuc,%	t1 nuc, ns	t2 nuc, ns	tau mean,ns	a1 cyto,%	t1 cyto,ns	t2 cyto,ns	tau mean cyto,ns
T24	0	81	0.35	2.51	0.75	77	0.38	2.65	0.89
	0.5	84	0.39	2.55	0.75	79	0.4	2.7	0.88
	1.5	89	0.36	2.39	0.59	82	0.37	2.65	0.75
	5	86	0.36	2.52	0.67	81	0.37	2.68	0.81
	24	87	0.36	2.42	0.63	81	0.37	2.63	0.79
Collagen +T24+huFB	0	88	0.36	2.42	0.61	83	0.38	2.67	0.75
	0.5	81	0.37	2.55	0.78	77	0.39	2.65	0.89
	1.5	84	0.36	2.5	0.7	80	0.37	2.63	0.84
	5	81	0.37	2.7	0.81	79	0.38	2.66	0.86
	24	82	0.35	2.48	0.74	78	0.37	2.61	0.85
Collagen +T24	0	86	0.39	2.4	0.7	82	0.4	2.72	0.82
	0.5	82	0.37	2.65	0.76	79	0.38	2.67	0.86
	1.5	85	0.36	2.48	0.67	80	0.37	2.65	0.82
	5	86	0.35	2.53	0.66	81	0.37	2.62	0.8
	24	81	0.37	2.59	0.78	78	0.38	2.72	0.91

MTT assay

In order to determine the origin of early metabolic rearrangements detected by NAD(P)H fluorescence, the MTT assay for metabolic activity was performed as the enzymatic reduction of MTT reagent to formazan is catalyzed by mitochondrial dehydrogenases, thus being dependent on mitochondrial respiration. By 5 hours of DOX exposure the optical density of formazan for T24 cells in collagen increased, indicating an activation of mitochondria. By 24 hours of DOX exposure the optical density decreased to 95.8±12.5 and 77.5±5.9% (p=0.000) for T24 cells with and without collagen correspondingly (Figure S8). The photomicrographs of formazan crystals showed differences in mitochondria organization in cells with and without collagen matrix. Before treatment, mitochondria in control T24 cells were organized into a fusioned network, while most cells in collagen had fragmented mitochondria. In 24 hours of DOX exposure the mitochondrial fission was seen in T24 cells without collagen. In contrast, the mitochondrial fusion was detected in T24 cells seeded in collagen after DOX exposure, which correlated with a higher mitochondrial activity (Figure S8). It is known that mitochondrial fusion supports increased OXPHOS, whereas mitochondrial fission is associated with decreased OXPHOS and has a protective role from mitochondrial ROS generation [58,59]. It is possible that the mitochondrial activation/fusion contributed to the higher survivability of cells in collagen upon treatment. Therefore, our results of FLIM of NAD(P)H are consistent with the assessment of mitochondrial structure.

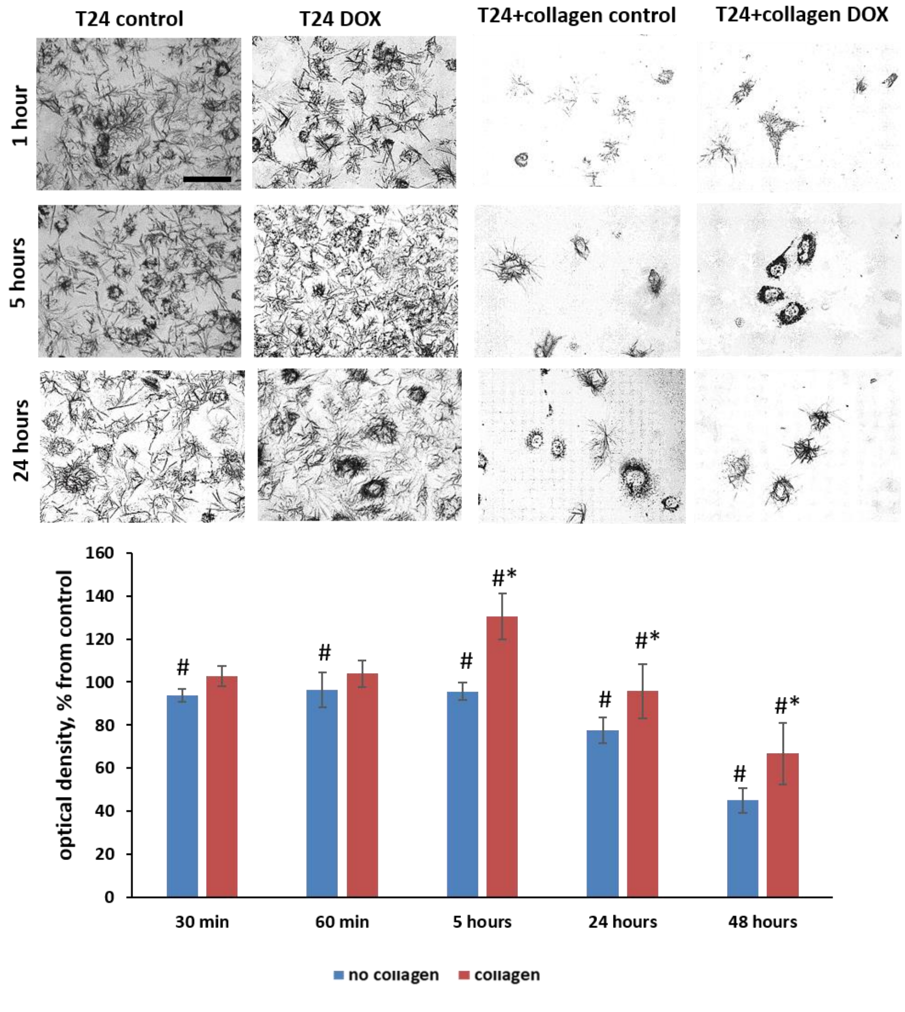

Figure S8. MTT-assay of T24 cells with and without collagen after treatment with DOX. White-field microscopy images and optical density obtained from formazan-stained cells after different time. Scale bar is 100 μm. Mean±SD. *Statistically significant difference from T24 cells without collagen at the selected point. #Statistically significant difference from the control value.
